# Supplementary material for: Immune Cell Deconvolution Reveals Possible Association of γδ T Cells with Poor Survival in Head and Neck Squamous Cell Carcinoma
Source: Cancers (Basel). 2023 Oct 5;15(19):4855. doi: 10.3390/cancers15194855 (PMC10571517; doi:10.3390/cancers15194855)
Supplement: Supplementary file 1 [file cancers-15-04855-s001.zip › cancers-2587211-supplementary.pdf]

SUPPLEMENTAL MATERIALS

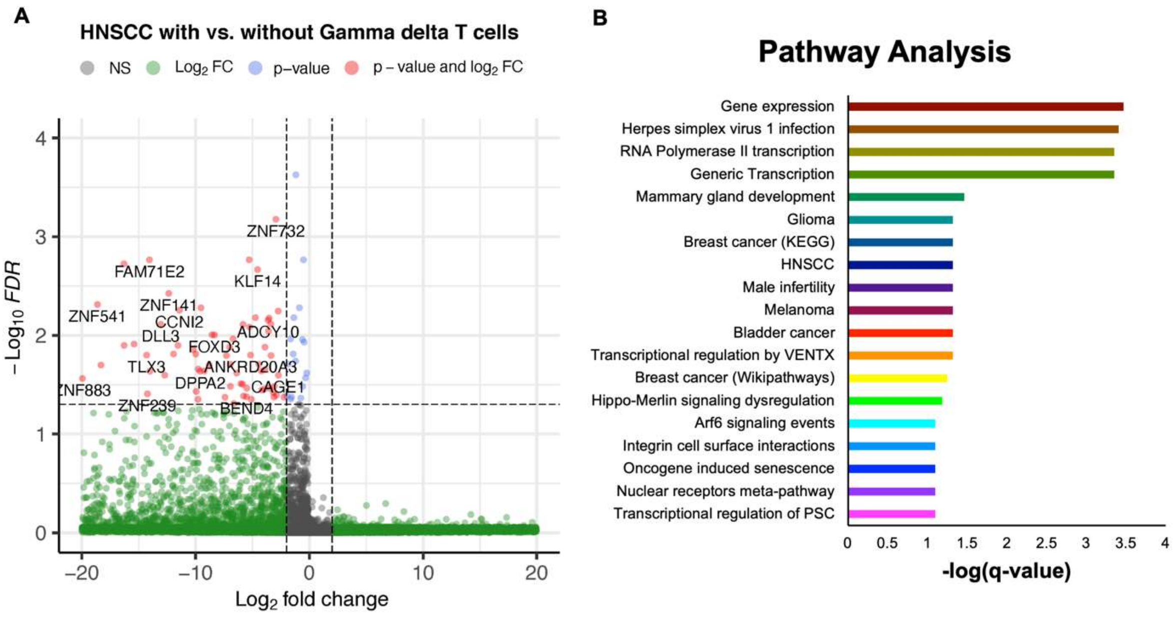

Supplementary Figure S1. Differential gene expression and pathway analysis in HPV-negative HNSCC with and without γδ T cells. (A) Volcano plot shows differential gene expression analysis. Genes to the right were more highly expressed in tumors with γδ T cells, while genes to the left were more highly expressed in tumors without γδ T cells. (B) Bar plot shows significance of pathways activated in tumors with γδ T cells.

Supplementary Table S1. Univariate Cox proportional hazards model of the association of 22 individual immune cell types in the LM22 matrix with disease free survival in HPV-negative HNSCC. Immune cell type level is as computed by CIBERSORT deconvolution.

Cell Type	Level	N (%)	P (log-rank)
B cells naïve	[0,0.0165]<br>(0.0165,0.248]	238 (75.1)<br>79 (24.9)	0.37
B cells memory	0<br>1	249 (78.5)<br>68 (21.5)	0.52
Plasma cells	[0,0.0388]<br>(0.0388,0.32]	238 (75.1)<br>79 (24.9)	0.85
T cells CD8	[0,0.0695]<br>(0.0695,0.362]	238 (75.1)<br>79 (24.9)	0.33
T cells CD4 naïve	0<br>1	306 (96.5)<br>11 (3.5)	0.47
T cells CD4 memory resting	[0,0.156]<br>(0.156,0.311]	238 (75.1)<br>79 (24.9)	0.09
T cells CD4 memory activated	[0,0.0112]<br>(0.0112,0.158]	238 (75.1)<br>79 (24.9)	0.02
T cells follicular helper	[0,0.0997]<br>(0.0997,0.254]	238 (75.1)<br>79 (24.9)	0.17
T cells regulatory	[0,0.0627]<br>(0.0627,0.159]	238 (75.1)<br>79 (24.9)	0.65
T cells $\gamma\delta$	0<br>1	303 (95.6)<br>14 (4.4)	0.008
NK cells resting	[0,0.0741]<br>(0.0741,0.202]	238 (75.1)<br>79 (24.9)	0.17
NK cells activated	[0,0.016]<br>(0.016,0.126]	238 (75.1)<br>79 (24.9)	0.93
Monocytes	[0,0.00748]<br>(0.00748,0.123]	238 (75.1)<br>79 (24.9)	0.11
Macrophages M0	[0,0.391]<br>(0.391,0.801]	238 (75.1)<br>79 (24.9)	0.37
Macrophages M1	[0,0.16]<br>(0.16,0.401]	238 (75.1)<br>79 (24.9)	0.24
Macrophages M2	[0,0.179]<br>(0.179,0.42]	238 (75.1)<br>79 (24.9)	0.66
Dendritic cells resting	[0,0.00872]<br>(0.00872,0.184]	238 (75.1)<br>79 (24.9)	0.47
Dendritic cells activated	[0,0.0536]<br>(0.0536,0.332]	238 (75.1)<br>79 (24.9)	0.66
Mast cells resting	[0,0.0402]<br>(0.0402,0.155]	238 (75.1)<br>79 (24.9)	0.09
Mast cells activated	[0,0.0231]<br>(0.0231,0.218]	238 (75.1)<br>79 (24.9)	0.50
Eosinophils	0<br>1	288 (90.9)<br>29 (9.1)	0.27
Neutrophils	[0,0.00117]<br>(0.00117,0.108]	238 (75.1)<br>79 (24.9)	0.55
**Total**	**--**	**317 (100)**	**--**

Supplementary Table S2. Clinicopathologic characteristics of patients with and without γδ T cells. No statistically significant differences were found, supporting the notion that the presence of γδ T cells is an independent prognostic factor.

	γδ T cells		
**Factor**	**Zero N (%)**	**Non-zero N (%)**	**P (chi-sq)**
**Gender**			
Male	210 (69.3)	8 (57.1)	
Female	93 (30.1)	6 (42.9)	0.51
**Smoking status**			
Never/former	189 (64.3)	12 (85.7)	
Current	105 (35.7)	2 (14.3)	0.17
**T stage**			
T1-T2	101 (34.7)	6 (42.9)	
T3-T4	190 (65.3)	8 (57.1)	0.74
**N stage**			
N0	140 (48.6)	6 (46.2)	
N+	148 (51.4)	7 (53.8)	0.99
**Overall stage**			
I	12 (4.1)	0 (0.0)	
II	61 (20.7)	4 (28.6)	
III	58 (19.7)	1 (7.1)	
IVA	154 (52.4)	9 (64.3)	
IVB	7 (2.4)	0 (0.0)	
IVC	2 (0.7)	0 (0.0)	0.72
**Perineural invasion**			
Negative	110 (49.5)	4 (36.4)	
Positive	112 (50.5)	7 (63.6)	0.59
**Lymphovascular invasion**			
Negative	143 (65.9)	9 (90.0)	
Positive	74 (34.1)	1 (10.0)	0.22
**Subsite**			
Oral Cavity	214 (70.6)	12 (85.7)	
Larynx	69 (22.8)	1 (7.1)	
Oropharynx HPV-negative/missing	20 (6.6)	1 (7.1)	0.38
**Subtype**			
Basal	89 (29.4)	5 (35.7)	
Atypical	54 (17.8)	1 (7.1)	
Classical	40 (13.2)	2 (14.3)	
Mesenchymal	120 (29.6)	6 (42.9)	0.78
**Total**	303	14	--

Supplementary Table S3. Pathway analysis of differentially expressed genes across tumors with and without γδ T cells.

q-value	Pathway	Source	Relevant genes
3.41E-04	Gene expression (Transcription)	Reactome	PIWIL2; ZNF75A; FOXG1; EGFR; AGRP; TNRC6A; RICTOR; ZNF141; ZNF140; ZNF169; ZNF354C; ITGA2B; ZNF620; ZNF621; BRPF3; ZNF782; TAF4B; NR5A1; ZNF764; ZNF558; ZNF184; CDKN2A; ITGA4; TDRKH; ZNF300; ZNF257; ZNF718; ZNF732; MGA; ESR2; ESR1; CCND1; TOP3A; TAF8; NUP153; ZNF514; NUP155; ZNF461; POU2F2
3.90E-04	Herpes simplex virus 1 infection - Homo sapiens (human)	KEGG	ZNF141; ZNF140; ZNF169; ZNF177; ZNF718; ZNF354C; ZNF184; ZNF879; ZNF620; ZNF621; ZNF257; ZNF300; ZNF782; ZNF514; ZFP82; ZNF764; ZNF461; POU2F2; ZNF558; ZNF814
4.44E-04	Generic Transcription Pathway	Reactome	ZNF75A; FOXG1; ITGA4; ZNF257; TNRC6A; RICTOR; ZNF141; ZNF140; ZNF169; ZNF354C; ITGA2B; ZNF620; ZNF621; BRPF3; ZNF782; TAF4B; NR5A1; ZNF764; ZNF514; ZNF184; CDKN2A; EGFR; ZNF300; AGRP; ESR2; ZNF732; MGA; ZNF718; ESR1; CCND1; ZNF558; ZNF461; TOP3A
4.44E-04	RNA Polymerase II Transcription	Reactome	ZNF75A; FOXG1; ITGA4; ZNF257; TNRC6A; RICTOR; ZNF141; ZNF140; ZNF169; ZNF354C; ITGA2B; ZNF620; ZNF621; BRPF3; ZNF782; TAF4B; NR5A1; ZNF764; ZNF514; ZNF184; CDKN2A; EGFR; ZNF300; AGRP; ESR2; ZNF732; MGA; ZNF718; ESR1; CCND1; TOP3A; TAF8; ZNF558; ZNF461; POU2F2
3.43E-02	Mammary gland development pathway - Pregnancy and lactation (Stage 3 of 4)	Wiki-pathways	CCND1; ESR2; EGFR; ESR1
4.77E-02	Transcriptional Regulation by VENTX	Reactome	CCND1; CDKN2A; TNRC6A
4.77E-02	Bladder cancer - Homo sapiens (human)	KEGG	CCND1; EGFR; CDKN2A; E2F3
4.77E-02	Melanoma - Homo sapiens (human)	KEGG	CCND1; EGFR; IGF1R; CDKN2A; E2F3
4.77E-02	Male infertility	Wiki-pathways	ESR2; PIWIL2; ESR1; TEX15; MLH3; AHRR; BRDT
4.77E-02	Head and Neck Squamous Cell Carcinoma	Wiki-pathways	CCND1; IGF1R; CDKN2A; EGFR; RICTOR
4.77E-02	Breast cancer - Homo sapiens (human)	KEGG	IGF1R; E2F3; ESR1; CCND1; EGFR; DLL3; ESR2
4.77E-02	Glioma - Homo sapiens (human)	KEGG	CCND1; EGFR; IGF1R; CDKN2A; E2F3
5.65E-02	Breast cancer pathway	Wiki-pathways	IGF1R; E2F3; ESR1; CCND1; EGFR; DLL3; ESR2

6.51E-02	Hippo-Merlin Signaling Dysregulation	Wiki-pathways	IGF1R; CCND1; EGFR; ITGA4; ITGA2B; ITGAD
7.95E-02	Transcriptional regulation of pluripotent stem cells	Reactome	SALL1; FOXD3; DKK1
7.95E-02	Nuclear Receptors Meta-Pathway	Wiki-pathways	B3GNT5; GSTT2; ESR1; CCND1; EGFR; AHRR; SERPINB9; ABCG8; SLC5A11; ZIC2
7.95E-02	Oncogene Induced Senescence	Reactome	CDKN2A; E2F3; TNRC6A
7.95E-02	Integrin cell surface interactions	Reactome	ITGAD; ICAM4; ITGA4; ITGA2B
7.95E-02	Arf6 signaling events	PID	ITGA2B; EGFR; TSHR
